# Age-Dependent Neuroimmune Modulation of IGF-1R in the Traumatic Mice

**DOI:** 10.1186/1742-4933-9-12

**Published:** 2012-05-28

**Authors:** Hui Zhao, Xiaocong Zhao, Xiaoding Cao, Gencheng Wu

**Affiliations:** 1 Department of Integrative Medicine and Neurobiology, National Key lab of Medical Neurobiology, Institute of Brain Research Sciences, Shanghai Medical College, Fudan University, Shanghai, People’s Republic of China; 2 Department of Integrative Medicine and Neurobiology, Shanghai Medical College, Fudan University, 138# Yixueyuan Rd., Box 291, Shanghai, 200032, People’s Republic of China

**Keywords:** IGF-1R, Fyn, Synapse, Neuroimmune modulation, MOR

## Abstract

**Background:**

Age-dependent neuroimmune modulation following traumatic stress is accompanied by discordant upregulation of Fyn signaling in the frontal cortex, but the mechanistic details of the potential cellular behavior regarding IGF-1R/Fyn have not been established.

**Methods:**

Trans-synaptic IGF-1R signaling during the traumatic stress was comparably examined in wild type, Fyn (−/−) and MOR (−/−) mice. Techniques included primary neuron culture, in vitro kinase activity, immunoprecipitation, Western Blot, sucrose discontinuous centrifugation. Besides that, [^3^ H] incorporation was used to assay lymphocyte proliferation and NK cell activity.

**Results:**

We demonstrate robust upregulation of synaptic Fyn activity following traumatic stress, with higher amplitude in 2-month mice than that in 1-year counterpart. We also established that the increased Fyn signaling is accompanied by its molecular connection with IGF-1R within the synaptic zone. Detained analysis using Fyn (−/−) and MOR (−/−) mice reveal that IGF-1R/Fyn signaling is governed to a large extent by mu opioid receptor (MOR), and with age-dependent manner; these signaling cascades played a central role in the modulation of lymphocyte proliferation and NK cell activity.

**Conclusions:**

Our data argued for a pivotal role of synaptic IGF-1R/Fyn signaling controlled by MOR downstream signaling cascades were crucial for the age-dependent neuroimmune modulation following traumatic stress. The result here might present a new quality of synaptic cellular communication governing the stress like events and have significant potential for the development of therapeutic approaches designed to minimize the heightened vulnerability during aging.

## Background

It has shown that surgery depresses several aspects of immune functions, including decreased splenocyte proliferation and natural killer cell activity [1], impaired T cell proliferation [2] and bactericidal activity [3], reduced production of a number of cytokines [4,5]. Aging was recently was identified as an exaggeration for several stress responses by decline of proteostasis, DNA damage repair networks and mitochondrial respiratory metabolism [6,7]. Our previous observation confirmed this realization that when challenged with traumatic stress, 1-year rats displayed deteriorated immuno-suppression and prolonged recovery than 3-month counterpart. The major hypothesis has been proposed Fyn, a member of Src-family protein tyrosine kinase, as an explanation, whose age-dependent expression was responsible for the related cellular responses induced by traumatic stress, and presumed to be crucial for the resolution of this stressful event [8].

It has been well defined that Fyn is localized to the lipid rafts microdomain, emerging as distinct entry portals of signal compartment and played an essential role in cell migration, proliferation, gene expression, metabolism, and cytoskeletal architecture. Specifically, Fyn was presumed to be required for activation of growth factors [9,10]. It is now firmly established that stress-induced immune activation is solely initiated by neuronal activity and probably involves efficient neural circuits within the lipid rafts, by which enhanced inhibitory synaptic drive. Particularly, related cell microenvironment is also modulated and amplified by aging, and several growth factors was involved in this process. For example, elevated IGF-1R signaling happens naturally in response to stressful environmental conditions [11,12], and is thought to promote cell maintenance and protection in aged life by catalyzing a physiological shift toward pathways involved Src kinase [13]. Then, the question is whether IGF-1R signaling could be triggered when challenged with traumatic stress?

For many years, opioid system arose as part of the immunomodulatory system [14]. MOR activation results in considerably reduction of macrophage and leukocytes functions [15-17]. MOR could also influence specific immunity by decreasing antibody production [18], lymphocyte proliferation and apoptosis [19]. Most important, opioid system plays an evolutionary role on maintaining dynamic equilibrium in stress by orchestrating endocrine, behavioral and autonomic responses [4,20,21]. For example, MOR expression in brain and immune organs was elevated after exposure to prolonged restraint stress [22,23]. Novel findings further provide information regarding the cross-talk of MOR and IGF-1R in the increased environmental stress resistance [24], Fyn within the lipid rafts might contribute to the higher susceptibility to their correlation [14], by which reinforcing IGF-1R activation in response to the the traumatic stress. Accordingly, we used Fyn (−/−) and MOR (−/−) mice in the present study, and predicted that MOR might be the cue for IGF-1R/Fyn activation. Furthermore, these signal cascades were mainly concentrated within synaptic zone, wherein compromised the cellular communication and appear to have wide appreciation for the mechanism underlying context-dependent microenvironment changes in terms of aging or stress like events.

## Results

### Induction of Fyn signaling in pre-and PSD fraction by traumatic stress

**Figure 1:**
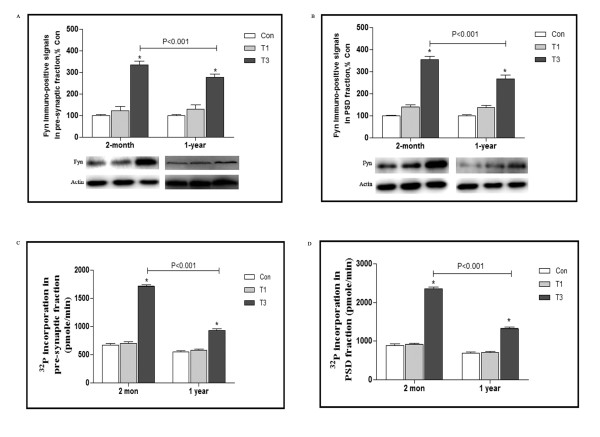
**Induction of Fyn signaling in pre-and PSD fraction by traumatic stress.** 2-month and 1-year mice were ere killed 1 and 3 days after traumatic stress (n = 5 for each group), pre-synaptic and PSD fraction from frontal cortex were prepared. Western blot analysis was used to detect Fyn and actin expression **(A and B)**. Data are normalized and presented as percentage of control, with the relative density of Fyn in the control group (without operation) set at 100%. Values represent mean ± SD for 3 independent experiments. *P <0.05 vs Con. The homogenates were immunoprecipitated using anti-Fyn (1:200), ^32^P incorporation in the resulting pellets were determined by incubating with 5 μg of Src substrate peptide in kinase buffer at 30°C, data was converted to pmol/min **(C and D)**. Values represent mean ± SD for 3 independent experiments. *P <0.05 vs Con. Con: control; T1 and 3: 1 and 3 days after trauma.

As described in our previous report, Fyn signaling in frontal cortex was essential for age-dependent neuroimmune modulation, then whether Fyn expression and activity was related with synaptic function? Firstly, two ages of mice were used and divided into 3 groups: Control (Con), 1 and 3 day after trauma (T1 and T3). Pre- and Post-synaptic density (PSD) fraction were separated by subcellular fractionation, Western Blot analysis using anti-Fyn antibody demonstrated a dynamic modulation of protein levels following trauma. Levels were continuously at low level in Con and T1 group, but strongly increased to 3.4 and 2.8 folds over control in 2-month and 1-year mice respectively. Intriguingly, parallel changes in PSD fraction were observed, immuno-positive densities for Fyn were progressively rose to 3.6 and 2.8 folds over control at day 3 following trauma in 2-month and 1-year mice (Figure 1A and B).

We further investigated if changes in Fyn expression are accompanied by parallel changes in Fyn activity during traumatic stress. As shown in Figure 1C and D, pre- and PSD fraction were separated from frontal cortex and immunoprecipitated by anti-Fyn antibody, Src kinase-catalyzed phosphorylation of synthetic target peptides using [γ-^32^P] ATP revealed that ^32^P incorporation in pre-synaptic fraction was remained at the low level until day 1 following trauma, thereafter it was considerably enhanced at day 3, levels were 2.5 and 1.7 folds over control in 2-month and 1-year mice respectively. Likewise, in PSD fraction, ^32^P incorporation showed similar alteration in response to traumatic stress, and also with higher magnitude in 2-month mice that in 1-year counterpart.

### Co-localization of IGF-1R and Fyn within synaptic zone during traumatic stress

**Figure 2:**
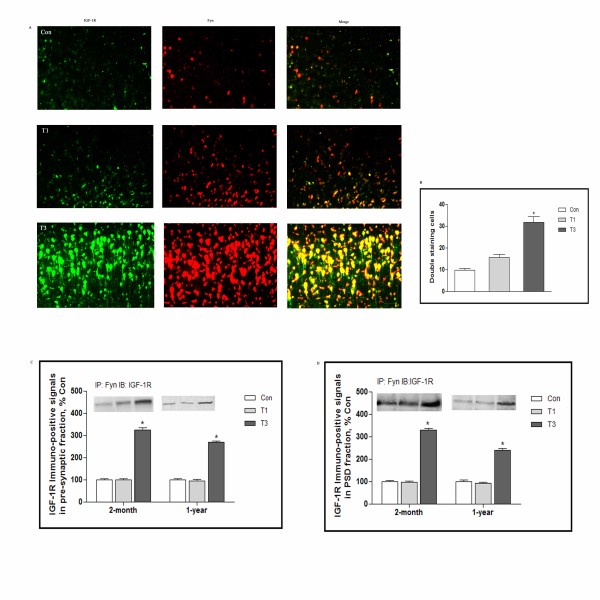
**Co-localization of IGF-1R and Fyn within synaptic zone during traumatic stress.** 2-month and 1-year mice were killed 1 and 3 days after traumatic stress (n = 5 for each group), cross section of frontal cortex were immunostained using anti-IGF-1R and anti-Fyn antibodies, and double-labeled cells were identified using a Leica Q500IW image analysis system **(A)**. The graph depicts expressions as percentages of controls **(B)**. Pre-synaptic **(C)** and PSD regions **(D)** were isolated, and association of IGF-1R with Fyn was determined by immunoprecipitation assay. The immunoprecipitation antibody was anti-Fyn and the immunoblotting antibody was anti-IGF-1R. Data were normalized and calculated as percentage of control, values represent mean±SD for 3 independent experiments. Con: control; T1 and 3: 1 and 3 days after trauma. *p<0.05 *vs* Con. Scale bars, 50μm.

We then examined the association of IGF-1R and Fyn following traumatic stress. 2-month mice were challenged with surgical trauma and analysis was also at days 1 and 3 after trauma. By fluorescent double staining, IGF-1R and Fyn immuno-positive signals were stained by green and red fluorescent respectively, the double staining cells were showed with yellow color. It was revealed that co-localization of IGF-1R and Fyn was gradually increased in frontal cortex, reaching a maximum at 3 days following trauma (Figure 2A and B).

We also detect the alteration of IGF-1R and Fyn interaction in response to traumatic stress in 1-year mice (data not shown), which showed similar change pattern with that in 2-month subjects, however, there were no detectable changes between these two age groups of mice, it is therefore mandatory to measure the age-dependent association of IGF-1R and Fyn association by immunoprecipitation. As shown in Figure 2C and D, immuno-positive signals for IGF-1R were robustly increased at day 3 following trauma when pre-synaptic fraction was pooled with anti-Fyn antibody, the expression levels were 3.3 and 2.7 folds of control in 2-month and 1-year mice respectively. Interestingly, in 2-month and 1-year mice, association of IGF-1R and Fyn in PSD fraction was also up-regulated, when immunoprecipitated with anti-Fyn antibody, immuno-positive signals for IGF-1R rose to 3.3 and 2.4 folds over control.

### Subcellular distribution of IGF-1R during traumatic stress

**Figure 3:**
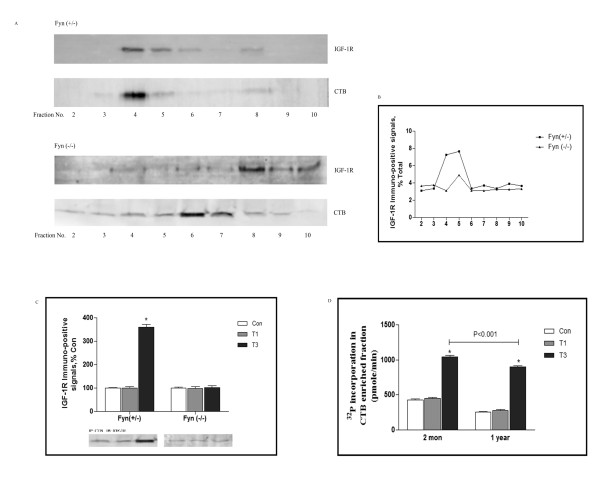
**Subcellular distribution of IGF-1R during traumatic stress.** Fyn (+/−) and Fyn (−/−) mice were killed 1 and 3 days after traumatic stress (n = 5 for each group), a lipid raft from frontal cortex was prepared to determine subcellular distribution of IGF-1R. Western blot analysis was used to detect IGF-1R expression in fractions 2–10, and CTB immunopositive fractions were identified as lipid raft fractions **(A)**. Data were calculated as percentage of total, each value represents mean± SD for 3 independent experiments **(B)**. Fyn (+/−) and Fyn (−/−) mice were killed 1 and 3 days after traumatic stress (n = 5 for each group), a lipid raft from frontal cortex was prepared, and association of IGF-1R with CTB was determined by immunoprecipitation assay. The immunoprecipitation antibody was anti-CTB and the immunoblotting antibody was anti-IGF-1R. Data were normalized and calculated as percentage of control, values represent mean±SD for 3 independent experiments **(C)**. 2-month and 1-year Fyn (+/−) were killed 1 and 3 days after traumatic stress (n = 5 for each group), a lipid raft from frontal cortex was prepared and immunoprecipitated using anti-Fyn (1:200), ^32^P incorporation in the resulting pellets were determined by incubating with 5 μg of Src substrate peptide in kinase buffer at 30°C, data was converted to pmol/min, values represent mean±SD for 3 independent experiments **(D)**. Con: control; T1 and 3: 1 and 3 days after trauma. *p<0.05 *vs* Con.

Lipid raft microdomain was currently believed to be a critical signal compartment for Fyn activity profile [25], we therefore detected subcellular distribution of IGF-1R in frontal cortex during traumatic stress. In Fyn (+/−) mice, by sucrose discontinuous centrifugation and Western Blot analysis, it was shown that the peak of IGF-1R immuno-positive signals were concentrated within fraction 4 and 5, the considered lipid raft microdomain in which enriched with cholera toxin B subunit (CTB) immuno-reactive signals. However, in Fyn (−/−) mice, immuno-positive signals for IGF-1R and CTB were widely distributed from fraction 2 to 9, there were no longer CTB enriched fraction exist (Figure 3A and B). To further confirm the distribution of IGF-1R within the lipid rafts, immunoprecipitation was used to measure the association of IGF-1R and CTB. As shown in Figure 3C, materials in fraction 4 and 5 were pooled by anti-CTB antibody, IGF-1R immuno-positive signals were considerably increased at day 3 following trauma, the binding interaction rose to 3.6 folds over control. Intriguingly, in Fyn (−/−) mice, there was not detectable alteration in anti-IGF-1R immunoprecipitates in CTB material following trauma (Figure 3C), indicating that distribution of IGF-1R within the lipid rafts was dependent on Fyn.

We then examined the role of Fyn in the lipid rafts related function. 2-month and 1-year mice were undergone traumatic stress, CTB enriched fraction from frontal cortex was analyzed by ^32^P incorporation for Fyn activity. As shown in Figure 3D, ^32^P incorporation was generally present at low levels in control group and day 1 following trauma, however, by day 3, it was predominantly upregulated, with higher magnitude in 2 month-mice than that in 1-year counterpart. Accordingly, our data are consistent with the possibility that Fyn was indeed crucial for the recruitment of signals into the lipid rafts.

### IGF-1R/Fyn signaling within neuronal lipid rafts

**Figure 4:**
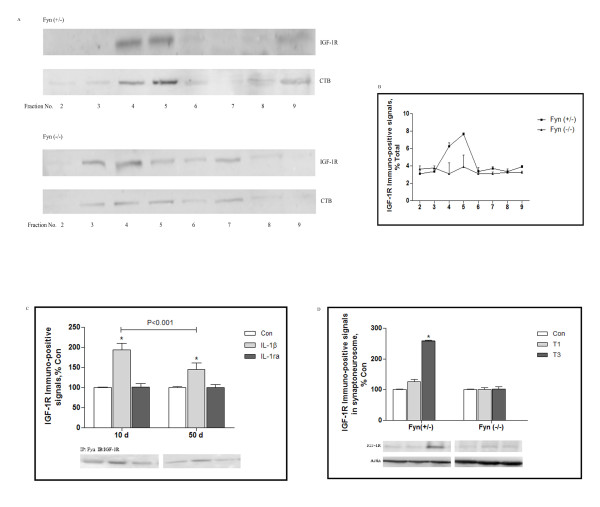
**IGF-1R/Fyn signaling within neuronal lipid rafts.** Cortical neurons from Fyn (+/−) and Fyn (−/−) mice were cultured for 10 days. A lipid raft was prepared to determine sucellular distribution of IGF-1R. Western Blot analysis was used to detect IGF-1R expression in fractions 2–9, and CTB immunopositive fractions were identified as lipid raft fractions **(A)**. Data were calculated as percentage of total, each value represents mean± SD for 3 independent experiments **(B)**. Cortical neurons from Fyn (+/−) mice were cultured for 10 and 50 days. Neurons were treated with vehicle or IL-1β, IL-1ra for the indicated time, and then assessed for the presence of IGF-1R in CTB material by immunoprecipitation. Data were normalized and calculated as percentage of control, each value represents mean± SD for 5 independent experiments **(C)**. Fyn (+/−) and Fyn (−/−) mice were killed Wild type and Fyn (−/−) mice were 1 and 3 days after traumatic stress (n = 5 for each group), synaptoneurosome in the frontal cortex were extracted, IGF-1R expression was measured by Western Blot analysis. Data were normalized and calculated as percentage of control, each value represents mean±SD for 3 independent experiments **(D)**. *p<0.05 *vs* Con. Con: control; T1 and 3: 1 and 3 days after trauma.

It was previously described that IGF-1R signaling was mostly associated with neurons [26], we then investigated subcellular distribution of IGF-1R in cortical neuron. As shown in Figure 4A and B, subcellular fractions from Fyn (+/−) neurons were analyzed by Western blot for IGF-1R, as expected, immuno-positive signals for IGF-1R were highly enriched in fraction 4 and 5, wherein harbored most proportion of CTB expression. However, in Fyn (−/−) neurons, immuno-positive signals for IGF-1R and CTB were generally present in the non-raft fractions. Lately, it was reported that long-term neuron culture could provide a useful tool for studies on neuronal development, aging, and neurotransmission [27], then we cultured neurons from Fyn (+/−) mice for 10 and 50 days, thereafter, the neurons were exposed to IL-1β, the central mediator of traumatic stress [4], molecular interaction of IGF-1R and Fyn was assayed by immunoprecipitation. Figure 4C illustrated that in control cells, IGF-1R immunopositivity in Fyn material was at low level, however, following IL-1β exposure, the proportion of IGF-1R was predominantly associated with Fyn, the binding interaction was increased to 2.0 and 1.5 folds over control in 10d and 50d cultures respectively, this effect was potently and specifically blocked by IL-1ra. Therefore, our data indicated that IGF-1R/Fyn signaling might decline along with cellular senescence.

To address the effect of neuronal IGF-1R/Fyn signaling on synaptic function, we determined to examine their association within synaptoneurosome when mice were challenged with traumatic stress. Figure 4D revealed that in Fyn (+/−) mice, IGF-1R expression was generally present at low in control group, then gradually increased, by day 3 following trauma, the expression levels were remarkably increased to 2.6 folds of control. Conversely, in Fyn (−/−) mice, IGF-1R expression did not show any difference during the traumatic stress. The data further reinforced the idea that IGF-1R/Fyn signaling within the synaptic zone could be age-dependently initiated by traumatic stress.

### Modulation of IGF-1R/Fyn signaling within the synaptic zone during traumatic stress

**Figure 5:**
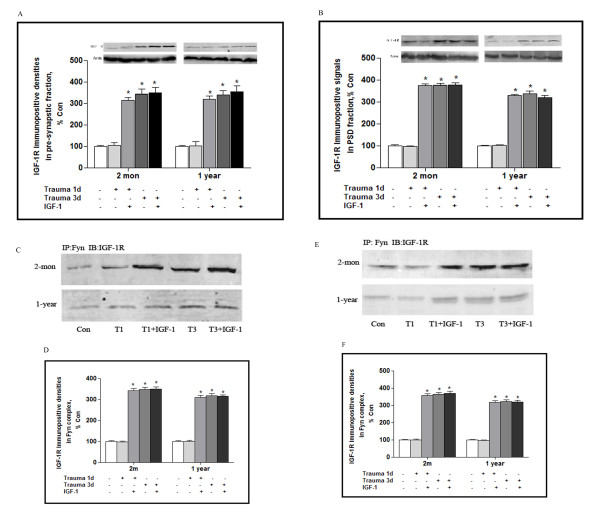
**Modulation of IGF-1R/Fyn signaling within the synaptic zone during traumatic stress.** 2-month and 1-year mice were subjected to surgical trauma, some of these mice were injected icv with IGF-1. Thus, 5 groups of mice were created: Con, T1, T1 + IGF-1, T3, T3 + IGF-1 (n = 5 for each group). Pre-synaptic and PSD fraction from frontal cortex were prepared and assessed for IGF-1R expression using Western Blot **(A and B)**. The interaction of IGF-1R and Fyn was assessed by immunoprecipitation **(C and E)**. Panel D and F depict quantitative analysis of C and E respectively. Data were calculated as percentage of control, each value represents mean±SD for 3 independent experiments **(D)**. *p<0.05 *vs* Con. Con: control; T1 and 3: 1 and 3 days after trauma.

These observations together suggest that IGF-1R/Fyn signaling is strongly upregulated by traumatic stress, it is therefore possible that these signaling cascades could be modulated in vivo. Accordingly, 2-month and 1-year mice were injected icv with IGF-1, and changes in levels of IGF-1R expression in pre-synaptic and PSD fraction were measured by Western blot. As shown in Figure 5A and B, at day 1 following trauma, IGF-1 resulted in a dramatic increase in levels of IGF-1R expression not only in pre-synaptic but also in PSD fraction, with higher level in 2-month mice than that in 1-year counterpart. For day 3 following trauma, IGF-1 only functioned to sustain the higher expression level.

We also explored the effects of IGF-1 on the association of IGF-1R and Fyn. As shown in Figure 5C and D, at day 1 following trauma, anti-Fyn immunoprecipitates of pre-synaptic fraction were robustly increased by IGF-1 administration, the binding interaction rose to 3.5 and 3.1 folds over control in 2-month and 1-year mice respectively. For day 3 following trauma, IGF-1 icv injection was also to sustain the close binding of IGF-1R and Fyn. Similarly in PSD fraction, IGF-1 administration augmented the interaction of IGF-1R and Fyn at day 1 following trauma, with higher magnitude in 2-month mice than that in 1-year subject. For day 3 following trauma, IGF-1R immuno-positive signals in Fyn material remained at high level after IGF-1 administration (Figure 5E and F).

### Involvement of IGF-1R/Fyn in the traumatic stress mediated neuroimmune modulation

**Figure 6:**
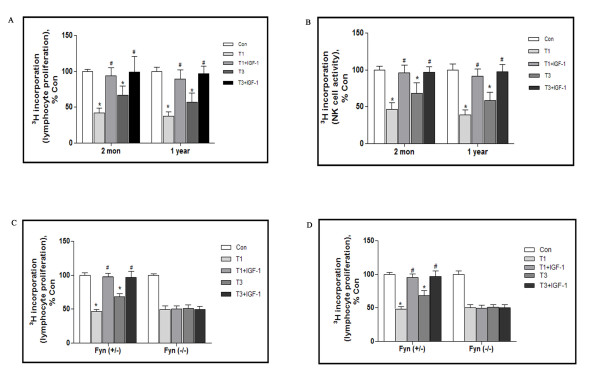
**Involvement of IGF-1R/Fyn in the traumatic stress mediated neuroimmune modulation.** 2-month and 1-year mice were subjected to surgical trauma, some of these mice were injected icv with IGF-1. Thus, 5 groups of mice were created: Con, T1, T1 + IGF-1, T3, T3 + IGF-1 (n = 5 for each group). Homogenates of spleen were prepared, and lymphocyte proliferation **(A)** and NK cell activity **(B)** were assayed by [^3^ H] incorporation. Fyn (+/−) and Fyn (−/−) mice were subjected to surgical trauma, some of these mice were injected icv with IGF-1. Thus, 5 groups of mice were created: Con, T1, T1 + IGF-1, T3, T3 + IGF-1 (n = 5 for each group). Homogenates of spleen were prepared, and lymphocyte proliferation **(C)** and NK cell activity **(D)** were assayed by [^3^ H] incorporation. Data are presented as percentage of control. Values represent mean ± SD for 3 independent experiments. *p < 0.05 vs Con, #*p* < 0.05 vs traumatized. Con: control; T1 and 3: 1 and 3 days after trauma.

Given that IGF-1R could be initiated by traumatic stress, there might have mechanistic link between IGF-1R and the related neuroimmune modulation. To address this possibility we studied the effects of IGF-1 on the suppression of lymphocyte proliferation and NK cell activity following traumatic stress. This revealed that IGF-1 administration led to significant increase in both lymphocyte proliferations and NK cell activity, [^3^ H] incorporation for lymphocyte proliferation was 93.5 ± 11.2 and 98.8 ± 21.8% of control at day 1 and 3 after trauma in 2-month mice, 89.4 ± 12.7 and 96.4 ± 10.5% of control in 1-year mice (Figure 6A). For NK cell activity, they were 95.8 ± 10.9 and 91.8 ± 9.5% of control at day 1 and 3 after trauma in 2-month mice, 96.6 ± 7.9 and 97.4 ± 9.8% of control in 1-year mice (Figure 6B). Equivalent modulation of IGF-1 administration on both age groups of mice might due to upregulation of Fyn signaling.

To investigate the potential involvement of Fyn in this process, Fyn (+/−) and Fyn (−/−) mice were used to address the link between IGF-1R/Fyn and neuroimmune modulation in traumatic mice. As shown in Figure 6C and D, IGF-1 exerted a similar progressive effect on lymphocyte proliferation and NK cell activity in Fyn (+/−) mice. However, in Fyn (−/−), abrogation of lymphocyte proliferation and NK cell activity were sustainable, IGF-1 administration could not rescue this immunosuppression.

### Initiation of IGF-1R/Fyn signaling by MOR in the traumatic mice

Because Fyn is known to specifically transmit MOR information [28], also, IGF-1R was hypothesized to be transactivated by MOR, we investigate if changes in IGF-1R/Fyn signaling are accompanied by parallel changes in MOR. Accordingly, MOR (+/−) and MOR (−/−) mice were undergone traumatic stress, synaptoneurosome in frontal cortex was prepared and assayed for Fyn activity. As shown in Figure 6 A, in MOR (+/−) mice, incorporation of ^32^P into the Src-specific substrate peptide was potentially strengthened at day 3 following trauma. Instead, in MOR (+/−) mice, there were no detectable changes in ^32^P incorporation during traumatic stress.

**Figure 7:**
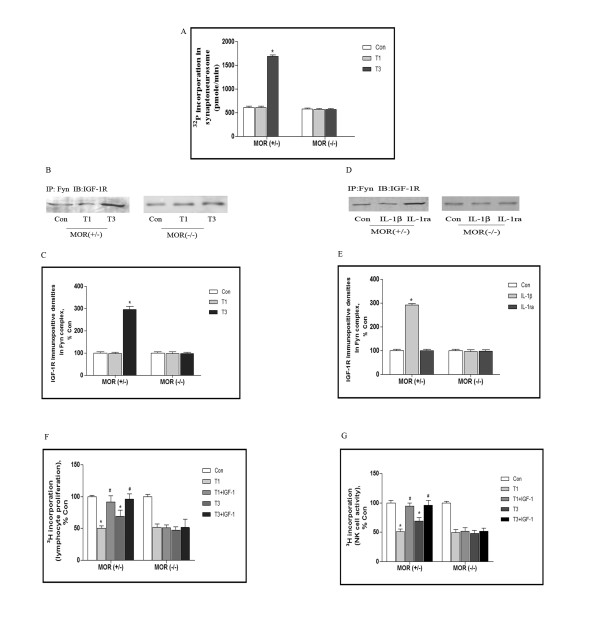
**Initiation of IGF-1R/Fyn signaling by MOR in the traumatic mice.** MOR (+/−) and MOR (−/−) mice were subjected to surgical trauma, some of these mice were injected icv with IGF-1. Thus, 5 groups of mice were created: Con, T1, T1 + IGF-1, T3, T3 + IGF-1 (n = 5 for each group). Synaptoneurosome in the frontal cortex was prepared and pooled with anti-Fyn antibody, ^32^P incorporation were determined by incubating with 5 μg of Src substrate peptide in kinase buffer at 30°C. Data was converted to pmol/min **(A)**. Synaptoneurosome was prepared and assayed for association of IGF-1R and Fyn by immunoprecipitation. Anti-Fyn was used as immunoprecipitate antibody and anti-IGF-1R as immuno blot antibody **(B)**. Panel C depicts quantitative analysis of B. Cortical neurons from MOR (+/−) and MOR (−/−) mice were cultured for 10 days, then exposed to IL-1β and IL-1ra respectively, association of IGF-1R and Fyn was determined by immunoprecipitation **(D)**. Panel E depicts quantitative analysis of D. MOR (+/−) and MOR (−/−) mice were subjected to surgical trauma, some of these mice were injected icv with IGF-1. Thus, 5 groups of mice were created: Con, T1, T1 + IGF-1, T3, T3 + IGF-1 (n = 5 for each group). Homogenates of spleen were prepared, and lymphocyte proliferation **(F)** and NK cell activity **(G)** were assayed by [^3^ H] incorporation. Data were normalized and calculated as percentage of control, each value represents mean±SD for 3 independent experiments. *p* < 0.05, *vs Con, ^#^*vs* traumatized. Con: control; T1 and 3: 1 and 3 days after trauma.

Moreover, association of IGF-1R and Fyn in frontal cortex was examined by immunoprecipitation, in which anti-Fyn antibody was used as immunoprecipitation antibody and anti-IGF-1R as immunoblot antibody. Figure 7B and C illustrated that in MOR (+/−) mice, the binding densities were greatly enhanced at day 3 following trauma, immuno-positive signals for IGF-1R in Fyn material rose to 3.0 folds over control. Conversely, in MOR (−/−) mice, we could not detect above alterations during traumatic stress. To confirm the effect of MOR on IGF-1R/Fyn signaling in vitro, we cultured cortical neurons from MOR (+/−) and MOR (−/−) mice. Figure 7C and D revealed that in neurons from MOR (+/−) mice, upon exposed to IL-1β, the binding densities for IGF-1R and Fyn were enhanced to 2.9 folds over control, the effect was specifically blocked by IL-1ra. However, in neurons from MOR (−/−) mice, IL-1β could exert its regulation on the interaction of IGF-1R and Fyn like that observed in neurons from MOR (+/−) mice.

We then examined if MOR was involved in neuroimmune modulation in the traumatic mice. As shown in Figure 7F and G, in MOR (+/−) mice, IGF-1 administration led to significant increase not only in lymphocyte proliferations but also in NK cell activity, [^3^ H] incorporation for lymphocyte proliferation was 91.3 ± 23.2 and 96.2 ± 19.1% of control at day 1 and 3 after trauma, for NK cell activity, they were 94.3 ± 13.2 and 95.2 ± 19.0% of control. nth mice, 96.6 ± 7.9 and 97.4 ± 9.8% of control in 1-year mice (Figure 6B). However, in MOR (−/−) mice, lymphocyte proliferation and NK cell activity were sustained at low level during traumatic stress, regardless of IGF-1 administration.

### MOR expression during traumatic stress

**Figure 8:**
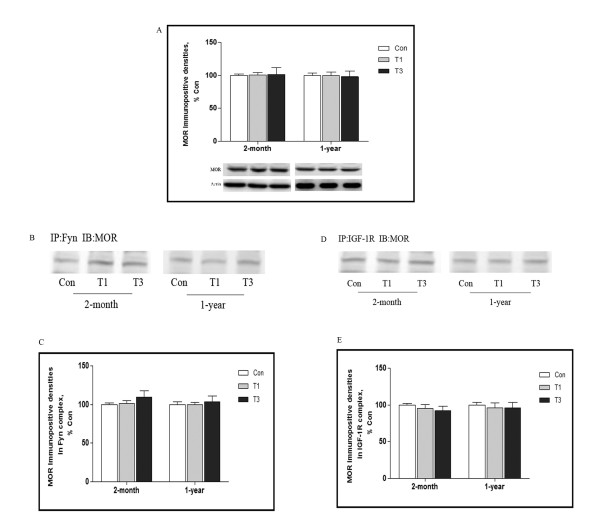
**MOR expression during traumatic stress.** 2-month and 1-year mice were killed 1 and 3 days after traumatic stress (n = 5 for each group), synaptoneurosome from frontal cortex was prepared, Western blot analysis was used to detect MOR expression **(A)**. Immunoprecipitation was used to analyze alterations of MOR and IGF-1R/Fyn interaction. The immunoprecipitation antibody was anti-Fyn **(B)** or anti-IGF-1R **(D)** and the immunoblotting antibody was anti-MOR. Panel C and E depict quantitative analysis of B and D respectively. Data are presented as percentage of control, values represent mean ± SD for 3 independent experiments. Con: control; T3: 3 days after trauma.

To address the question that the increased IGF-1R/Fyn signaling is due to MOR itself or the downstream signaling, finally, we determined to detect MOR expression during traumatic stress. As shown in Figure 8A, MOR expression within frontal cortical synaptoneurosome was assayed by Western Blot, there was not detectable alteration in MOR immuno-positive signals during traumatic stress, no matter in 2-month or 1-year mice. Association of MOR with IGF-1R, or MOR with Fyn was further assayed by immunoprecipitation, anti-IGF-1R or anti-Fyn was used as immunoprecipitation antibody and anti-MOR as immunobloting antibody, Figure 8B and C illustrated that both in 2-month and 1-year mice, MOR immuno-positive signals in Fyn enriched pool were remained at the same level during. Coincidently, there were no detectable changes in MOR immuno-positive signals in IGF-1R enriched pool during traumatic stress either (Figure 8D and E). Then, our data indicated that IGF-1R/Fyn was probably triggered by MOR downstream signaling, by which conveying signals for age-dependent neuroimmune modulation.

## Discussion

Many studies documented that stress resistance could be extended by aging, even mild or optimal insults can evoke sustained stress and cause detrimental changes in aged subjects [29,30]. Ultimately, reductions in synaptic numbers and the amplitude of the field excitatory postsynaptic potential (EPSP) happened during the aging [31]. In the present study, we demonstrated that synaptic Fyn activity was greatly augmented at day 3after trauma, with higher magnitude in 1-year mice that that in 2-month counterpart, together with our previous observation, namely, discordant Fyn signaling events was responsible for the deteriorated immuno-suppression and prolonged recovery aged rats during the traumatic stress [8,32], then, the result reinforced the idea that Fyn signaling might actively communicate with pre and post-synaptic elements of neuronal terminals influencing stress like events.

We have already known that, In the central nervous system (CNS), Fyn controls switches in a variety of signal transduction pathways governing cell growth, division, differentiation, and survival [33] by assembling a unique platform, lipid rafts [34]. It has also been noted that the principle of Fyn activation appears to be realized by its affinity with receptors for growth factors, especially, IGF-1R, and the specialized cellular communication within the lipid rafts. Then firstly, we found that in regards with traumatic stress, Fyn was predominantly coupled to IGF-1R functioning as central elements in synaptic zone. Secondly, we demonstrated that IGF-1R is expressed at high levels and exists in a structurally distinct and activated form within lipid rafts derived from synaptoneurosome, ultimately, this recruitment is account of coupling with Fyn, and are better able to be initiated by traumatic stress with age-dependent manner. Since there are studies showing the significant role of lipid rafts related signal transduction in the synaptic function, and lipid raft-localized signaling molecules is more catabolically active than non-raft localized one [35,36], thus, we proposed that discordant functional IGF-1R/Fyn signaling is dispensable for the preservation of synaptoneurosome in certain cellular properties, which potentially leading to age-related alterations when confronted with traumatic stress.

Then, the question is how the IGF-1R/Fyn signaling was differentially switched on when challenged with traumatic stress? It was evident that IGF-1R could be modulated by IGF-1. Upon icv injection of IGF-1, IGF-1R/Fyn signaling within the synaptic zone was considerably augmented, the effects were not only in 2-month mice, but also in 1-year mice. When dissociated with Fyn, IGF-1R signaling will be defected. Furthermore, as demonstrated, IGF-1R/Fyn signaling was able to rescue the immuno-suppression mediated by traumatic stress in Fyn (+/−) mice but not in Fyn (−/−) mice. Since IGF-1R signaling was found to be correlated with longevity, and could extend life span by more than 2-fold [37,38]. Then, our present data provided further evidence that IGF-1R/Fyn might present a new quality of signaling, by which provoked trans-synaptic cellular communication when challenged with the traumatic stress [39]. Also, the age-dependent provoking of IGF-1R and Fyn during the traumatic stress might extensively indicate their connection with the sustained or detrimental stress-related cellular behaviors in aged subjects.

From the pharmacological receptor theories, we learned that the stimulation of a receptor must not inevitably lead to the switch on of all downstream signaling pathways, that the incomplete activation of signaling may also occur [40-42]. Very recently, it was hypothesized that IGF-1R transactivation has been mostly related with the stimulation of a particular GPCR. Since Fyn could specifically transmit MOR information, which ultimately recruit more protein kinases to the lipid rafts and lead to an array of neuronal responses [43-46]. Intriguingly, MOR activation was usually resulted in attenuated anti-CD3/CD28, stimulated IFN-γ promoter activity and bimodal modulation of LPS-induced expression of IL-6 and TNF-α [17,47-49]. Then, we assumed that MOR is one of the crucial elements for triggering IGF-1R signaling when challenged with traumatic stress. As demonstrated, Fyn activity, as well as its molecular connection with IGF-1R was dependent on MOR, on account of that their coupling was obviously not able to be observed when lack of MOR, accordingly, there was not improvement from the immuno-suppression mediated by traumatic stress in MOR (−/−) mice. Likewise, there was not remarkable change in MOR expression, as well as the association of MOR with IGF-1R or Fyn in the synaptic zone. Then, it is plausible that a characteristic feature of IGF-1R was probably due to age-dependent Fyn activation that was triggered by MOR signaling cascades, this specialized process was mainly concentrated within synaptic zone and might contribute to the recovery from traumatic stress mediated immuno-suppression.

## Conclusions

Indeed, we still lack of the evidence regarding the direct phosphorylation of IGF-1R by Fyn or IGF-1R trafficking during stress like events. But, we have shown that IGF-1R signaling events required Fyn-related lipid rafts as a signaling platform for the assembly of signaling molecules. Most importantly, age-dependent feature of IGF-1R was delivered probably by Fyn via MOR signaling cascades. Therefore, it has been proposed that the maintenance of these trans-synaptic signaling cascades could be an important determining factor for preserving synaptic functions minimizing the heightened vulnerability of the brain in aging, especially when confronted with traumatic mice.

## Methods

### Traumatic animal model

Mice including C57BL/6 J (Animal center of Fudan University Shanghai Medical College), MOR (−/−) and Fyn (−/−) mice (2 month old, on a C57BL/6 background, Department of Pharmacology, University of Minnesota) were used in the present study. Dorsomyotomy and exploratory laparotomy were performed on mice under anesthesia (sodium pentobarbital 3.5 mg/100 g, i.p.) as the model of traumatic stress. No post operative infection occurred. Tissue samples were taken 1 day after operation. All protocols were approved by the Committee on Research Animal Care of Fudan University, and the principles and procedures outlined in the NIH Guide for the Care and Use of Laboratory Animals were observed.

### Intracerebroventricular injection of drugs

Implantation of the cannula was performed stereotaxically under anesthesia, the stainless steel guide cannula (0.5 mm in diameter) with an inserted cannula (0.25 mm in diameter) was implanted into the right lateral ventricle (posterior 0.5, lateral 1.5, horizontal 4.5) and fixed on the skull with dental cement. Mouse recombinant IGF-1 (Thermo Fisher Scientific Inc. Rockford, IL, 5 μg) dissolved in sterilized PBS was injected over 10 s via the cannula at a volume of 10 μl. Mice from the control group were injected with vehicle. At the end of each experiment, the position of the cannula was assessed by histological examination. Only data collected from experiments in which correct insertion of the cannula was verified were reported. Animals were killed 2 hours after IGF-1 injection.

### Immunal assay

For lymphocyte proliferation, spleens were pressed through stainless steel mesh and red blood cells were lysed by treatment with NH_4_Cl solution. Then cell suspension of 1 × 10^7^ cells/ml in a final volume of 200 μl of complete tissue culture medium (RPMI 1640 supplemented with 10% heatinactivated fetal calf serum, 2 mM L-glutamine) was seeded in triplicate in a U-bottom 96-well plate in the presence and absence of concanavalin A (Con A, 1 mg/L). Plates were incubated at 37°C in a 5% CO_2_. After 48 h, cultures were labeled with 0.5 μCi of [^3^ H]thymidine (Amersham Biosciences, Buckinghamshire, UK) and after 24 h, cells were harvested using a cell harvester. Samples were counted in a liquid scintillation counter. Proliferation results are presented as the mean cpm ± SEM of triplicate cultures.

For natural killer cell cytotoxicity, firstly suspensions of YAC-1 lymphoma cells with a concentration of 2 × 10^5^/ml at a final volume of 100 μl were targeted with 0.5 μCi of [^3^ H] thymidine and incubated at 37°C, 5% CO2 for 6 h. Then spleens were homogenized and the resultant cell suspensions were pooled in the presence and absence of Con A and seeded in triplicate with the effector: target ratios of 50:1 for 16 h. Cytotoxic activity results were determined as follows:

Percent response + [(counts in tested well-counts in spontaneous response well)/(counts in maximum response well-counts in spontaneous response well)]×100.

### Neuronal cell culture (long-term neuron culture)

Cortical neurons were from embryonic day 16 mice. Cortices were dissected and collected, fetuses were decapitated and cortical tissue was collected under sterile conditions. After removing meninges, cortical tissue was dissociated in 0.05% trypsin at 37°C. Dissociated neurons were washed in DMEM and gently suspended in neuron-defined serum-free Neurobasal medium supplemented with B27. Neurons were cultured for 10 and 50 days, then treated with indicated drugs: IGF-1 (R&D systems, Minneapolis, MN; 100 ng/ml) for 30 min; IL-1β (R&D systems, Minneapolis, MN; 20 ng/ml, 24 h) and IL-1ra (R&D systems, Minneapolis, MN; 10 ng/ml, 24 h).

### Immuno-fluorescent double-labeling

Mice were anesthetized with sodium pentobarbital (3.5 mg/100 g, i.p.) and transcardially exsanguinated with 0.1 M PBS followed by perfusion of the fixation (4% paraformaldehyde in 0.1 M PBS, pH7.4), each provided in a 7 ml/min flow rate. Serial sets of 20 μm coronal sections from brains were collected on a freezing microstome (Leica, SM2000R). Frozen sections were subjected to IGF-1R (Abcam, San Francisco, CA, 1:500) and Alexa 488 conjugated secondary antibody (Abcam, San Francisco, CA, 1:1000), anti-Fyn (BD transduction laboratories, 1:100) and Alexa 594 conjugated secondary antibody (Abcam, 1:1000) fluorescent antibodies respectively. The data derived from each group were analyzed by Leika Q500IW image analysis system. Frontal cortex was chosen for analysis and immuno-positive cells were semi-quantified under photomicrography.

### Coimmunoprecipitation and Src kinase activity

Proteins were extracted in buffer containing 20 mM HEPES (pH 7.5), 10 mM potassium chloride, 1.5 mM magnesium chloride, 1 mM ethylenediaminetetraacetic acid, 1 mM EGTA, and 13 complete protease inhibitor (Roche Applied Science). The sample was centrifuged at 10,000 g for 15 min at 48 C, and the supernatant was incubated with anti-Fyn (1:200) on a rotating platform overnight, followed by incubation with 20 μl protein G agarose beads (Pierce Biotechnology, Rockford, IL) for 2 h at 48 C. For Western Blot analysis, beads were washed three times in lysis buffer, and proteins were extracted and resolved in SDS-polyacrylamide gel and transferred to a polyvinylidene difluoride membrane (PVDF, Amersham Biosciences, Piscataway, NJ). The membrane was probed with anti-MOR (Neuromics, Edina, MN, 1:200) or IGF-1R (1:500), and subsequent alkaline phosphatase-conjugated secondary antibody (1:5000). The bands were detected by ECF substrate (Amersham Biosciences, Piscataway, NJ) and were quantified using ImageQquant software.

For Src kinase activity, beads were washed three times in lysis buffer and incubated at 30°C with 5 μg of Src substrate peptide (KVEKIGEGTYGVVYK, corresponding to amino acids 6–20 of p34^cdc2^; Upstate) in kinase buffer containing 5 μCi of [γ-^32^P]ATP (Perkin Elmer Life Sciences, San Jose, CA), 50 mM Tris–HCl, pH 7.5, 10 mM MgCl2, 10 mM MnCl_2_, 25 μM ATP, 1 mM dithiothreitol, and 100 μM Na_3_VO4. 30 min later the reaction was terminated by the addition of 10μl of 40% TCA, and the sample was spotted onto P81 cellulose phosphate paper (Upstate, Boston, MA). The paper was washed three times with 1% phosphoric acid and one time with acetone. Radioactivity retained on the P81 paper was quantified by liquid scintillation counting. Blank counts (without tissue lysate) were subtracted from each result, and radioactivity (cpm) was converted to picomoles per minute (pmol/min).

### Subcellular fractionation

Isolation of a presynaptic and PSD fraction was performed essentially as described previously [26,27]. Frontal cortex were collected and homogenized in 3 ml of 0.32 M sucrose, 0.1 mM CaCl2, with 30 μl each of protease inhibitor cocktail and phosphatase inhibitor cocktail (Sigma, St. Louis, MO) at 4°C. All of the following fractionation steps were carried out at 4°C unless otherwise specified. The homogenate was brought to a final concentration of 1.25 M sucrose by the addition of 2 M sucrose (12 ml) and 0.1 mM CaCl2 (5 ml). The homogenate was then placed in a 40 ml ultracentrifuge tube and overlaid with 10 ml 1 M sucrose, 0.1 mM CaCl2. The gradients were centrifuged at 100,000 g for 3 hrs. The synaptosomal fraction (4–5 ml) was collected at the 1.25 M/1 M interface. This fraction was then brought to a volume of 35 ml with 20 mM Tris-Cl pH 6, 0.1 mM CaCl2, containing 1% Triton X-100 (TX-100) and 350 μl of protease inhibitor cocktails, mixed for 20 min, and centrifuged at 40,000 g for 20 min. The pellet was collected to further separate PSD and pre-synaptic fraction.

The pellet was resuspended in 20 ml of 20 mM Tris-Cl pH 8, 1% TX-100, 0.1 mM CaCl2. The mixture was again mixed for 20 min, and centrifuged at 40,000 g for 20 min. The insoluble pellet containing the PSD fraction was collected and stored at −80°C until use. The supernatant was removed and concentrated to 1 ml using an Amicon Ultra-15 filter (Millipore, Bedford, MA). The concentrate was precipitated with 9 ml of acetone by incubation at −20°C for 12 hrs, and centrifugation at 15,000 g for 30 min. The resulting pellet, containing the pre-synaptic fraction, was stored at −80°C until use. Protein concentration was determined using the Bio-Rad Protein Assay (Bio-Rad Laboratories Ltd, Hayward, CA).

Frontal cortex was homogenized in 70 μl of ice cold Krebs-Henseleit (KRBS) buffer: 118.5 mM NaCl, 4.7 mM KCl, 1.18 mM MgSO4, 2.5 mM CaCl2, 1.18 mM KH2PO4, 24.9 mM NaHCO3, 10 mM dextrose, 10 μg/ml adenosine deaminase, pH7.4, 350 μl of protease inhibitor cocktails. The homogenate was diluted with 350 μl of additional ice-cold buffer. This mixture was loaded into a 1 ml Tuberculin syringe attached to a 13 mm diameter Millipore syringe filter holder. The diluted filtrate was forced over three layers of nylon (Tetko, 100 μm pore size) pre-wetted with 150 μl of KRBS, and collected in a 1.5 ml Eppendorf tube. The filtered particulate was then spun at 1000 g for 15 min in a microfuge at 4°C. The resultant pellet, containing synaptoneurosome fraction, was stored at −80°C until use Protein concentration was determined using the Bio-Rad Protein Assay (Bio-Rad Laboratories Ltd, Hayward, CA).

### Statistical analyses

All experiments were performed using 5 animals per group (n = 5). Data were represented as mean±SEM and analyzed with Prism 5 software. For all data sets, normality and homocedasticity assumptions were reached, validating the application of the one-way ANOVA, followed by *t* test for multiple comparison. Differences were considered significant for *p* < 0.05.

## Competing interests

The authors declare that they have no competing interests.

## Authors’ contributions

HZ produced the hypothesis for this study. XZ was responsible for the animal study. XC and GW gave extensive advice on the study. All authors read the manuscript, studied it critically for its intellectual content and approved the final draft.

## Funding

The study is supported by National Key Basic Research program of China (2009CB522900).
